# No Tryptophan, Tyrosine and Phenylalanine Abnormalities in Children with Attention-Deficit/Hyperactivity Disorder

**DOI:** 10.1371/journal.pone.0151100

**Published:** 2016-03-03

**Authors:** Catharina Elisabeth Bergwerff, Marjolein Luman, Henk J. Blom, Jaap Oosterlaan

**Affiliations:** 1 Clinical Neuropsychology section, Vrije Universiteit Amsterdam, Amsterdam, the Netherlands; 2 Center for Pediatrics and Adolescent Medicine, Medical Center–University of Freiburg, Freiburg, Germany; 3 Department of Clinical Chemistry, VU University Medical Center Amsterdam, Amsterdam, the Netherlands; Chiba University Center for Forensic Mental Health, JAPAN

## Abstract

**Background:**

The aim of the current study was to explore the role of aromatic amino acids (AAAs) in blood in relation to attention-deficit/hyperactivity disorder (ADHD). Given their impact on the synthesis of serotonin and dopamine, decreased concentrations of the AAAs tryptophan, tyrosine and phenylalanine in blood may contribute to the expression of ADHD symptoms. Decreased AAA blood concentrations, in turn, may be related to lowered dietary protein intake or to abnormal AAA catabolism, as evidenced by increased urinary AAA concentrations.

**Methods:**

Eighty-three children with ADHD (75% males) and 72 typically developing (TD) children (51% males), aged 6 to 13 years, participated in the study. AAA concentrations were assessed in blood spots and an 18-hour urinary sample. A nutritional diary was filled out by parents to calculate dietary protein intake. Parent and teacher questionnaires assessed symptoms of ADHD, oppositional defiant disorder, conduct disorder, and autism spectrum disorder.

**Results:**

Children with ADHD showed normal AAA concentrations in blood spots and urine, as well as normal protein intake compared to controls. No associations between AAA concentrations and symptoms of ADHD or comorbid psychiatric disorders were found.

**Conclusions:**

This study is the first to explore AAA metabolism in children with ADHD using a well-defined and relatively large sample. We found that AAA deficiencies are not related to ADHD. The results do not support treatment with AAA supplements in children with ADHD. Future studies regarding the cause of serotonin and dopamine alterations in ADHD should focus on other explanations, such as effects of altered transport of AAAs.

## Introduction

Attention-deficit/hyperactivity disorder (ADHD) is a childhood psychiatric disorder characterized by a persistent pattern of age-inappropriate inattention and/or hyperactivity-impulsivity [1]. Several risk factors have been proposed for the disorder, including environmental [2] and genetic [3] factors. Environmental factors include, among others, dietary abnormalities and psychosocial adversity, although odds ratios obtained for these risk factors are small or not significant [2]. Currently one of the main theories on genetic risk factors for ADHD involves aberrant dopaminergic neurotransmission [4, 5]. Dopamine receptor and transporter genes play a significant role in ADHD [6, 7], which may explain decreased dopamine levels in ADHD [8]. Other genetic studies provide evidence for an association between the serotonergic system and ADHD [9–11], in line with aberrant postsynaptic serotonin levels found in some individuals with ADHD [12]. Abnormal functioning of the dopamine and serotonin system has also been associated with neurocognitive deficits found in ADHD, such as cognitive impulsivity and poor executive attention [8]. Similarly, dopamine and serotonin abnormalities have been associated with psychiatric disorders that are highly comorbid with ADHD, including oppositional defiant disorder (ODD; [13]), conduct disorder (CD; [14]) and autism spectrum disorder (ASD; [15]).

While dopamine and serotonin hypotheses dominate current scientific work into ADHD, candidate gene study results are conflicting and effect sizes are small [3]. In addition to genetic risks of altered functioning of the neurotransmitter transporters and receptors, a potential interesting line of research focuses on the biosynthesis of dopamine and serotonin. Dopamine and serotonin are synthesized from aromatic amino acids (AAAs); the AAAs tyrosine and phenylalanine are precursors of dopamine and the AAA tryptophan is required for the synthesis of serotonin. While there are many other factors that affect the synthesis of dopamine and serotonin (including the transport of AAAs through the blood-brain barrier, and the availability of co-enzymes), normal circulating blood concentrations of AAAs are a first prerequisite for the synthesis of these neurotransmitters [16, 17]. Amino acids are a constituent of protein in foods, such as meat, bananas and milk [18, 19]. Phenylalanine and tryptophan are both essential AAAs, and therefore must be obtained by dietary means, but tyrosine can also be synthesized in the body from phenylalanine [20]. A lowered ingestion of protein or a malabsorption of AAAs may cause a decreased availability of AAAs [19]. In the current study we explore the hypothesis that decreased AAA blood concentrations contribute to ADHD symptom expression, assuming a relation between AAA blood concentrations and aberrant neurotransmission of dopamine and serotonin in ADHD.

Thus far, five case-control studies have been published on AAA blood concentrations in individuals with ADHD [21–25]. Three studies, of which two describing the same sample [21, 22], reported lower plasma concentrations of tryptophan, tyrosine and phenylalanine in ADHD [21–23]. The other two studies, however, showed increased concentrations of free tryptophan in ADHD [24] and a trend towards increased serum concentrations of tryptophan in children with ADHD [25]. All five studies are limited by non-standardized assessments of ADHD and small sample sizes (ranging from N = 12 to N = 48), and therefore further research into the availability of AAAs in ADHD is warranted. If blood concentrations of AAAs are decreased in an ADHD sample, this may be caused by reduced protein intake, malabsorption or increased catabolism of AAAs. Although there is little evidence for dietary abnormalities in ADHD [2], thus far no studies have specifically examined protein intake in ADHD. Increased urinary concentrations of AAAs may be indicative of an abnormal catabolism [26, 27] and four studies have investigated this hypothesis in small ADHD samples [21, 22, 28, 29]. While there is no evidence of abnormal levels of urinary tyrosine and phenylalanine concentrations in ADHD [21, 22, 28], one study showed increased urinary tryptophan concentrations, suggesting abnormal AAA catabolism [29]. Taken together, the currently available studies provide some evidence for an altered AAA availability in ADHD, although more research, with greater sample sizes and standardized procedures to assess ADHD, is required to gain more insight into the potential contribution of AAAs to the expression of ADHD symptoms.

The hypothesis that AAA concentrations are related to ADHD symptoms is the basis for a number of depletion and supplementation studies. Depletion of dietary tryptophan was found to impair sustained attention in adults with ADHD [30], and to weaken behavioural inhibition in hostile children with ADHD [31]. Supplementation with tryptophan, on the other hand, resulted in a decrease of ADHD symptoms in children with ADHD [32]. Tyrosine supplementation decreased ADHD symptoms in adults with ADHD [33], but showed no effects on behavioural functioning in children with ADHD [32]. Phenylalanine supplementation in adults with ADHD caused a decrease of restlessness and an increase on the ability to concentrate at trend level [34], but in children no effects were reported for phenylalanine supplementation on ADHD symptoms [35]. However, also these depletion and supplementation studies are limited by non-standardized assessments of ADHD and small sample sizes (ranging from N = 10 to N = 20), as well as the lack of control groups, hampering conclusions regarding the relation between AAAs and ADHD. Therefore, there is a need of further research to support the hypothesis that AAA concentrations may contribute to the expression of ADHD symptoms.

Another aspect that requires further research, is the association between AAAs and symptoms of childhood psychiatric disorders that are highly comorbid with ADHD. As pointed out, dopamine and serotonin abnormalities have also been associated with ODD, CD and ASD. Indeed, tryptophan depletion have been shown to induce aggressive behaviour [36, 37], and increased tryptophan levels have been found associated with childhood ASD [38], suggesting that AAA abnormalities might contribute to the expression of symptoms of ODD, CD and ASD. Given the heterogeneous evidence of AAA abnormalities in ADHD, comorbid psychiatric conditions might act as possible confounding (mediating) or exacerbating (moderating) factors, and should therefore be taken into account when studying AAA concentrations in ADHD.

To summarize, there is inconsistent evidence that AAAs, acting as precursors of dopamine and serotonin, contribute to the expression of ADHD symptoms. The mostly outdated studies on this topic performed thus far are hampered by methodological shortcomings. Therefore, our aim was to explore concentrations of tryptophan, phenylalanine and tyrosine in a well-phenotyped sample of children with ADHD as compared to a control sample consisting of typically developing (TD) children. We firstly hypothesized that children with ADHD would show decreased blood concentrations of tryptophan, tyrosine and phenylalanine compared to controls, and that below average AAA concentrations would increase the risk of being diagnosed with ADHD. Secondly, we hypothesized that blood AAA concentrations would be related to ADHD symptoms. Thirdly, we hypothesized that abnormal blood AAA concentrations would be related to a decreased protein ingestion or by an aberrant AAA catabolism as evidenced by increased urinary AAA concentrations. Finally, we studied the possible confounding effects of symptoms of ODD, CD and ASD on our findings.

## Materials and Methods

### Participants

Subjects were 83 children with ADHD (75% males) and 72 TD children (51% males), aged between 6 and 13 years. Inclusion criteria for the ADHD group were: (a) a clinical diagnosis of ADHD according to DSM-IV criteria, (b) confirmation of this diagnosis by the Diagnostic Interview Schedule for Children, fourth edition, administered to parents (DISC-IV-P; [39]), (c) significant ADHD symptoms, as indicated by parent ratings >90^th^ percentile on at least one of the ADHD scales (Inattention and Hyperactivity/Impulsivity scales) of the Disruptive Behaviour Disorder Rating Scale (DBDRS; [40]), and (d) pervasive ADHD symptoms, as indicated by teacher ratings >75^th^ percentile on at least one of the ADHD scales of DBDRS. Having a comorbid diagnosis (for example ODD or ASD) was no exclusion criterion, neither was treatment with stimulant medication. Children on stimulant medication (N = 50, 60% of the ADHD group) discontinued drug use 24 hours before testing, in order to allow complete washout [41], and during participation in our study. Inclusion criteria for the TD group were: (a) absence of a clinical diagnosis of any developmental or behavioural disorder (including ADHD and ODD), and (b) scores <90^th^ percentile on both parent- and teacher-rated ADHD scales of the DBDRS.

### Materials

#### Behaviour

Parents of children eligible for inclusion in the ADHD group were assessed with the disruptive behaviour disorder section of the DISC-IV-P. The DISC-IV-P is a widely used standardized diagnostic interview for the assessment of DSM-IV childhood psychiatric disorders, with adequate psychometric properties [39].

Parents and teachers of children in both the ADHD and TD group completed the DBDRS to assess ADHD symptoms and symptoms of ODD and CD. The DBDRS contains four scales measuring symptoms of inattention, hyperactivity/impulsivity, ODD and CD on a 4-point Likert scale (ranging from 0 to 3), with higher scores indicating worse symptoms. Adequate psychometric properties have been reported for the DBDRS [42].

The Strengths and Weaknesses of ADHD-symptoms and Normal Behaviour rating scale (SWAN; [43, 44]) was completed by parents and teachers to assess symptoms of ADHD. This widely used questionnaire contains two subscales; the Inattention scale and the Hyperactivity/Impulsivity scale, each comprising 9 items. Items are scored on a 7-point Likert scale (ranging from -3 to +3), with higher scores indicating worse symptoms. The items are based on the DSM-IV symptoms of ADHD, but reflect both ends (strong and weak) of the behaviour described in each ADHD symptom. Mean scores on both subscales were used as dependent variables. Adequate psychometric properties have been reported for the SWAN [45].

ASD symptoms were assessed using the 65–item Social Responsiveness Scale (SRS; [46, 47]), completed by parents and teachers. The items of the SRS are based on the DSM-IV symptom domains of ASD, including impairment in social interaction, communicative deficits and restricted/stereotypic patterns of behaviours or interests. The SRS uses a 4–point Likert scale (ranging from 0 to 3), and the summed item score on the total SRS scale was used as dependent measure, with higher scores indicating worse symptoms. The SRS has adequate psychometric properties [46, 48].

#### Blood spots

To investigate blood concentrations of tryptophan, tyrosine and phenylalanine, a dried blood spot technique was used. Collecting blood spots is less invasive for children than taking venous blood samples and the dried blood spot technique is sufficiently robust and stable for diagnostic purposes [49–51]. Blood spot AAA concentrations are highly correlated with serum AAA concentrations (*r*s ranging from .86 to .96) [52]. A blood spot of each child was collected using a disposable safety lancet. Three blood drops were spotted onto a blood stain card. A 5.5mm punch of a dried blood spot was mixed with 100μl of an internal standard solution (containing 29μM L-phenylalanine-D5, 6μM L-tyrosine-D4 and 5μM L-tryptophan-D5) and 400μl methanol in a Gas Chromatography vial (GC-vial) and shaken for 15 minutes in an ultrasonic bath. The supernatant was transferred in another GC-vial and evaporated under nitrogen at 30°C. Subsequently, the sample was butylated with 100μl of 5.5% acetyl chloride (in n-butanol) at 60°C for 15 minutes. Afterwards, the butanol-layer was evaporated under nitrogen (at 30°C) and the residue was dissolved in 500μl acetonitrile. Blood spot concentrations of tryptophan, tyrosine and phenylalanine were determined by positive electrospray liquid chromatography–tandem mass spectrometry (LC-MS/MS), using an API 3000 triple quadrupole mass spectrometer (Applied Biosystems, Foster City, CA, USA), coupled to a high-performance liquid chromatography (HPLC) system (Perkin Elmer Series 200, Shelton, USA). Three μl of the sample was injected on a symmetry C18 column (3.9*150mm, 5μm; Waters, Milford, MA, USA) and eluted with a flow rate of 1ml/min of 75% acetonitrile (containing 0.4% of formic acid). Tryptophan, tyrosine and phenylalanine eluted within 1 minute and were measured using the transitions: mass-to-charge ratio (m/z) 261.2→159.2 (tryptophan), m/z 238.2→136.2 (tyrosine) and m/z 222.2→120.2 (phenylalanine). All obtained LC-MS/MS data were acquired and processed using Analyst 1.4.2 software (Applied Biosystems, Foster City, CA, USA). The blood spot concentrations of tryptophan, tyrosine and phenylalanine were expressed in μmole/L. Reliability of the LC-MS/MS was confirmed by examining the inter-assay variance (being 5–10%), intra-assay variance (being 8–10%) and recovery (being 90–112%).

#### Dietary protein intake

Daily protein intake was assessed during three days, using a parent-reported nutritional diary. Standardized dietary records and instructions were provided. Parents were instructed to register all consumed foods and drinks in the dietary record and to express the consumed amounts as accurate as possible. The amount of protein intake (grams/day) was calculated based on a computerized version of the Dutch food composition database [53]. The Dutch food composition database contains over 2000 food products with information about the nutritional composition of these food products [54]. The database is widely used for scientific purposes (e.g.[55, 56, 57]).

#### Urine

In order to examine urinary AAA concentrations, participants collected all urine excreted within 18 consecutive hours (after-school hours) in a urine collection container. During urine collection, the container was stored in a refrigerator (<5°C). A 10ml sample was sent to a laboratory for analysis, where the sample was stored at -20°C. An HPLC technique with fluorescence detection was used for the analysis of tryptophan in urine [58]. The concentrations of tyrosine and phenylalanine in urine were determined using a Biochrom amino acid analyzer [59]. The urinary concentrations of tryptophan, tyrosine and phenylalanine were expressed by a μmole to total urine volume ratio, to rule out effects of polyuria or oliguria. Reliability of the HPLC technique was confirmed by examining the precision of the analyses, being 2.25% for tryptophan (relative standard deviation), and 1.50% for tyrosine and phenylalanine. There are high correlations between the amino acid concentrations in 12-hour samples and 24-hour samples [60], indicating that there is no diurnal variation in the excretion of amino acids, validating the use of an 18-hour sample in the current study.

### Procedure

This study received approval from the local medical ethical committee of the VU University Medical Center Amsterdam, the Netherlands (#NL39922.029.12), and has been performed in accordance with the ethical standards as laid down in the 1964 Declaration of Helsinki and its later amendments. Written informed consent was obtained of parents of all children, and of children ≥12 years, prior to participation. Children with ADHD were recruited from mental health outpatient clinics, through the parental association for children with behavioural problems, and through a university research website. The TD group was recruited from primary schools located throughout the country. Children on stimulant medication discontinued drug use one day prior to participation (day 0), to ensure complete washout, and during the assessment of blood, urine and food intake (day 1 to day 3). On day 1 the blood spot was collected in the early morning, to rule out the effects of diurnal variation of blood AAA concentrations. The same day, after school time, urine collection started and continued for the following 18 hours, until the child would return to school the next morning (day 2). In the early morning of day 1 parents received detailed instructions on how to fill out the dietary record and how to collect urine of their child. After the instruction, parents started recording their child’s dietary intake, which continued for the following three days (day 1 to day 3). Parents and teachers were invited to fill out the questionnaires on a secured website. All data were collected between February 2013 and July 2014. The ADHD and TD group were recruited simultaneously, to control for possible seasonal effects on food intake or AAA metabolism.

### Data analysis

All statistical analyses were performed using R, version 3.2.1. All variables were inspected on outliers and missing values for the ADHD and TD group separately. Winsorising was applied to outliers, these were replaced with a value one unit bigger (or smaller) than the previous most extreme score in the distribution of the group [61]. Missing data in the urinary concentrations, dietary data and behavioural data were randomly distributed and replaced using group means. For the blood spots there were no missing data. All data were normally distributed, except for CD symptoms. Group differences in gender were examined using a chi-squared test, and group differences in age and behavioural functioning were examined using independent samples t-tests.

To test the first hypothesis, group differences in AAA blood spot concentrations were assessed using Analyses of Variances (ANOVAs) with group (ADHD or TD) as fixed factor. Effect sizes were calculated in terms of partial eta squared, and interpreted as small (> .01), medium (>.06) or large (>.14) [62]. In addition, odds ratios were calculated, which expressed the risk for being diagnosed with ADHD with below average AAA concentrations. Normative data for AAA concentrations were derived from a large sample of 6 to 13 years old children (N = 104, 52% males) (unpublished data, sample information and results available from the authors). For each AAA, concentrations corresponding to the lowest 16^th^ percentile (M-1 SD) of the normative sample were used as a threshold value to define below average AAA concentrations (for tryptophan 45 μmole/L, tyrosine 39 μmole/L, and phenylalanine 47 μmole/L). Odds ratios were calculated with their 95% confidence interval and Fisher’s Exact Test was performed to examine the significance of the odds ratios.

To test the second hypothesis, Pearson product-moment correlation coefficients investigated the relationship between blood spot AAA concentrations and both parent and teacher rated symptoms of ADHD. The magnitude of correlation coefficients was interpreted as small (>.10), medium (>.30) or large (>.50) [62]. Data of the ADHD group and TD group were combined to maximize variability in the ADHD symptom measures.

To test the third hypothesis, correlation analyses between blood spot AAA concentrations and protein ingestion and urinary AAA concentrations were performed in the whole sample. We also examined whether there were group differences in protein ingestion and urinary AAA concentrations using ANOVAs with group (ADHD or TD) as fixed factor. Lastly, correlational analyses evaluated whether blood spot AAA concentrations were related to parent- and teacher-reported symptoms of comorbid psychiatric disorders (Pearson product-moment correlation coefficients for ODD and ASD, Spearman’s rank correlation coefficients for CD). If symptoms of ODD, CD or ASD were found related to the AAA concentrations, previous analyses were rerun with these symptoms entered as covariates. To correct for multiple testing, the alpha level of the correlation analyses was adjusted according to the Bonferroni procedure per outcome domain; ADHD symptoms (12 analyses, thus *p* = .004), potential determinants of AAA abnormalities in blood spots (12 analyses, thus *p* = .004), and symptoms of comorbid psychiatric disorders (18 analyses, thus *p* = .003). Bonferroni adjusted results are reported.

## Results

No groups differences were found in terms of age, but groups differed in gender as well as symptoms of ADHD, ODD, CD and ASD, see Table 1. The ADHD group had a larger proportion of males and more parent- and teacher-rated symptoms of ADHD, ODD, CD and ASD than the TD group. The DISC-IV-P indicated that in our ADHD sample, 65 children met DSM-IV criteria for the combined subtype of ADHD, 12 children met DSM-IV criteria for the predominantly inattentive subtype, and six children met DSM-IV criteria for the predominantly hyperactive-impulsive subtype.

**Table 1:** Group characteristics of the ADHD group (N = 83) and TD group (N = 72).

	ADHD group	TD group	Statistic
	*M (SD)*	*M (SD)*	
Age in months	116.71 (19.86)	119.17 (20.69)	t = -.75, NS
Males *N (%)*	62 (74.70)	37 (51.39)	χ^2^ = 9.08**
Parent-rated symptoms			
Inattention^a^	17.47 (4.82)	3.31 (3.07)	t = 22.11**
Hyperactivity/Impulsivity^a^	16.31 (5.97)	3.26 (2.71)	t = 17.92**
Inattention^b^	1.20 (.80)	-.46 (.72)	t = 13.64**
Hyperactivity/Impulsivity^b^	1.30 (.90)	-.39 (.90)	t = 11.58**
ODD^a^	9.72 (4.93)	3.04 (2.71)	t = 10.63**
CD^a^	2.45 (2.46)	.49 (.84)	t = 6.83**
ASD^c^	61.77 (25.93)	29.26 (14.23)	t = 9.84**
Teacher-rated symptoms			
Inattention^a^	14.71 (6.23)	1.85 (2.29)	t = 17.49**
Hyperactivity/Impulsivity^a^	13.80 (7.39)	1.57 (2.29)	t = 14.30**
Inattention^b^	1.04 (.85)	-.74 (1.02)	t = 11.66**
Hyperactivity/Impulsivity^b^	1.08 (.95)	-.83 (1.08)	t = 11.60**
ODD^a^	7.82 (6.04)	.89 (1.93)	t = 9.89**
CD^a^	2.20 (3.03)	.18 (.66)	t = 5.93**
ASD^c^	85.58 (21.84)	25.07 (14.18)	t = 20.71**

*Notes*.

^a^ Disruptive Behaviour Disorder Rating Scale,

^b^ Strengths and Weaknesses of ADHD-symptoms and Normal Behaviour rating scale,

^c^ Social Responsiveness Scale.

**p < .01. ADHD, Attention-Deficit/Hyperactivity Disorder; ASD, Autism Spectrum Disorder; CD, Conduct Disorder; NS, not significant; ODD, Oppositional Defiant Disorder; TD, Typically Developing

No significant group differences were observed for blood spot concentrations of tryptophan, tyrosine, or phenylalanine, see Table 2 (all *F*s<4.00 and *p*s>.05). A below average (<16^th^ percentile) blood spot concentration of phenylalanine increased the risk of being diagnosed with ADHD by 2.2, although this effect just escaped conventional levels of significance (*OR* = 2.22, 95%CI [.92–5.73], *p =* .07). A below average blood spot concentration of tryptophan (*OR* = 2.09, 95%CI [.86–5.40], *p =* .11) or tyrosine (*OR* = 1.83, 95%CI [.74–4.79], *p =* .22) did not increase the risk of being diagnosed with ADHD.

**Table 2:** Blood spot and urine concentrations of AAAs and protein ingestion in the ADHD and TD group.

	ADHD group (N = 83)	TD group (N = 72)	Statistic	
	*M (SD)*	*M (SD)*	*F*	p*η*^*2*^
*Blood spots*				
Tryptophan (μmole/L)	52.10 (10.06)	53.54 (9.10)	.87, NS	<.01
Tyrosine (μmole/L)	50.37 (15.92)	55.33 (15.88)	3.75, NS	.02
Phenylalanine (μmole/L)	56.40 (14.01)	57.46 (11.10)	.27, NS	<.01
*Dietary intake*				
Protein intake (g/day)	65.57 (16.05)	63.72 (13.86)	.58, NS	<.01
*Urine*				
Tryptophan (μmole/total urine)	38.09 (17.23)	38.26 (17.02)	< .01, NS	<.01
Tyrosine (μmole/total urine)	73.93 (33.25)	66.11 (29.72)	2.35, NS	.02
Phenylalanine (μmole/total urine)	48.36 (17.90)	46.74 (20.78)	.27, NS	<.01

*Notes*. AAA, Aromatic Amino Acid; ADHD, Attention-Deficit/Hyperactivity Disorder; NS, not significant; TD, Typically Developing

In the combined group of children with ADHD and TD children, AAA blood spot concentrations were not significantly related to ratings of ADHD symptoms (all *r*s < .24 and *p*s>.004). Furthermore, blood spot concentrations of tryptophan, tyrosine or phenylalanine were not significantly related to protein intake or urinary AAA concentrations (all *r*s < .19 and *p*s>.004). There were no differences between the ADHD and TD group with regard to protein intake or to urinary concentrations of tryptophan, tyrosine, or phenylalanine, see Table 2.

No significant associations between AAAs and symptoms of comorbid psychiatric disorders were found (all *r*s < .20 and *p*s>.003) and therefore none of the previous analyses were repeated adjusting for the effects of symptoms of ODD, CD or ASD. Since the ADHD group consisted of considerably more males than the TD group, the groups analyses were rerun with gender as a covariate. These analyses did not alter the results.

## Discussion

The main objectives of this study were to examine whether children with ADHD had decreased AAA blood spot concentrations, and whether blood spot AAA concentrations were related to symptoms of ADHD. In contrast to our hypothesis and some earlier studies on this topic [21–23], we did not find any differences in the AAA blood spot concentrations between the ADHD and TD group or associations between AAA blood spot concentrations and symptoms of ADHD. The finding that AAA alterations are not related to ADHD, argues against nutritional interventions with amino acid supplements for children with ADHD. In past years, some studies have examined the effects of AAA supplementation in children and adults with ADHD, with inconsistent results [28, 32–34]. The apparent lack of AAA deficiencies in ADHD might explain the conflicting results of amino acid supplementation on reducing ADHD symptoms [63]. It might be that the association between AAA blood concentrations (being precursors of serotonin and dopamine) and the expression of ADHD symptoms is too indirect to detect. There are several other factors involved in the metabolism of dopamine and serotonin that could be aberrant in ADHD. It might be that an altered transport of tryptophan across the blood-brain barrier [64] or an abnormal reuptake of dopamine and serotonin [12], moderate the association between AAA concentrations in blood and ADHD symptoms. Another explanation might be that AAA concentrations should be below a certain threshold before affecting the behaviour and functioning of children. In depletion studies, tryptophan concentrations drop by 60 to 90 percent [65] and tyrosine and phenylalanine concentrations by 74 percent [66], and are therefore much lower than baseline AAA concentrations, as measured in the current study. Clearly, the studies that focused hitherto on baseline concentrations of AAAs in ADHD were scarce and the results were inconsistent. Given our larger sample size, careful screening and correction for multiple comparisons, the current results challenge the hypothesis of AAA abnormalities in ADHD. Future studies regarding the serotonin and dopamine hypothesis in ADHD [12] may focus on other aspects of the serotonin and dopamine metabolism, such as the transport of AAAs [64], rather than on decreased AAA concentrations in blood.

We did not find evidence for altered protein intake in ADHD or for an association between protein intake and blood spot AAA concentrations. Further, we did not find any evidence for an aberrant AAA excretion, since no increased urinary AAA concentrations were found in children with ADHD and urinary AAA concentrations were not significantly related to blood spot AAA concentrations, in line with the results of previous studies on urinary AAA concentrations in ADHD [21, 22, 28]. Therefore, we believe that abnormal AAA metabolism, due to a failure of the intestines to absorb AAAs into the bloodstream or increased excretion into the urine, are no plausible causes of ADHD symptoms. Lastly, we did not find a confounding role of ODD, CD or ASD symptoms in the association between AAAs and ADHD.

There are some limitations to the current study that should be noted. Firstly, we measured AAA concentrations in blood and urine, which represent only two factors in the metabolic pathway of serotonin and dopamine. Therefore, we can only draw conclusions regarding the presence of AAAs in the blood, which is a first prerequisite for an adequate biosynthesis of serotonin and dopamine. Secondly, while our study is the largest of its kind and had an adequate power to detect medium-sized effects, our study was not sufficiently powered to detect small-sized effects. We found a relatively high odds ratio of 2.2 for being diagnosed with ADHD when having low phenylalanine concentrations, but this result just escaped conventional levels of significance. Therefore, our study does not definitively rule out that low phenylalanine concentrations are present in (a subgroup of) children with ADHD. For instance, it might be that only children with severe deficiencies in executive functioning have decreased phenylalanine concentrations, as an altered dopamine functioning in the prefrontal cortex and the striatum is thought to impair executive functions, including sustained attention and interference control in ADHD [8, 67]. Despite the limitations, our study is the first to explore AAA concentrations in children with ADHD using a well-defined and relatively large sample, showing that AAA abnormalities are not related to ADHD.
